# Effects of Music Training on the Auditory Working Memory of Chinese-Speaking School-Aged Children: A Longitudinal Intervention Study

**DOI:** 10.3389/fpsyg.2021.770425

**Published:** 2022-01-26

**Authors:** Peixin Nie, Cuicui Wang, Guang Rong, Bin Du, Jing Lu, Shuting Li, Vesa Putkinen, Sha Tao, Mari Tervaniemi

**Affiliations:** ^1^Cicero Learning, Faculty of Educational Sciences, University of Helsinki, Helsinki, Finland; ^2^Cognitive Brain Research Unit, Faculty of Medicine, University of Helsinki, Helsinki, Finland; ^3^State Key Laboratory of Cognitive Neuroscience and Learning and IDG/McGovern Institute for Brain Research, Beijing Normal University, Beijing, China; ^4^HiperCog Group, Department of Education, Faculty of Educational Sciences, University of Helsinki, Helsinki, Finland; ^5^Turku PET Centre, University of Turku, Turku, Finland; ^6^Turku University Hospital, Turku, Finland; ^7^Advanced Innovation Center for Future Education, Beijing Normal University, Beijing, China

**Keywords:** second-language training, music, training effect, transfer, randomized controlled trial, propensity score method

## Abstract

Music expertise is known to be beneficial for cognitive function and development. In this study, we conducted 1-year music training for school children (*n* = 123; 7–11 years of age before training) in China. The children were assigned to music or second-language after-class training groups. A passive control group was included. We aimed to investigate whether music training could facilitate working memory (WM) development compared to second-language training and no training. Before and after the training, auditory WM was measured via a digit span (DS) task, together with the vocabulary and block tests of the Wechsler Intelligence Scale for Child IV (WISC-IV). The results of the DS task revealed superior development in the music group compared to the other groups. However, further analysis of DS forward and backward tasks indicated that the performance of the three training/non-training groups only differed significantly in DS backward scores, but not in the DS forward scores. We conclude that music training may benefit the central executive system of WM, as reflected by the DS backward task.

## Introduction

The effects of music expertise beyond music/sound-related skills have been increasingly investigated since the 1990s. Studies suggest that individuals with music exposure perform better in tasks measuring language abilities, such as foreign language pronunciation skills (Milovanov et al., 2010), phonological awareness (Linnavalli et al., 2018), and verbal intelligence (Moreno et al., 2012) than those who without music exposure. In addition to these transfer effects on linguistic function, associations between music exposure and higher-level cognitive abilities, which may indicate far-transfer effects, have also been reported, for example, in non-verbal intelligence (Schellenberg, 2006) and academic skills (dos Santos-Luiz et al., 2016).

Despite these findings, the existence and interpretation of these far-transfer effects remain unclear. One perspective is that music lessons may enhance general cognitive abilities (e.g., WM and executive functions), and these abilities may mediate the amount of benefit received from music lessons to music-unrelated performance (Hannon and Trainor, 2007; Moreno and Bidelman, 2014). In other words, the high demands on listening, attention, and controlling behavior during the music learning process may facilitate domain-general executive functions. Prior studies have provided numerous findings regarding the effects of music training on cognitive functions (working memory: Roden et al., 2012; attention: Strait et al., 2015; executive functions: Degé et al., 2011; Slevc et al., 2016; for review: Talamini et al., 2017). However, the findings are inconsistent to some extent across different studies and measurements. Talamini et al. (2017) summarized thirty-seven studies in their meta-analysis and revealed a small effect size (*g* = 0.29) for long-term memory, a medium effect size (*g* = 0.57) for short-term memory, and a medium effect size (*g* = 0.56) for WM. Sala and Gobet (2017), in their meta-analysis, also reported a small effect size (*d* = 0.34) of music training on memory-related abilities, and the effect size was even smaller if random allocation of participants was conducted (Sala and Gobet, 2020). Recently, Bigand and Tillmann (2021) repeated Sala and Gobet’s (2020) analysis using the same data file, which resulted in stronger and more significant results. Here, we focus on music training’s effects on auditory WM, which has been viewed as predictive of other cognitive functions, such as general fluid intelligence and cognitive flexibility (Kane et al., 2004; Blackwell et al., 2009). Some researchers have proposed that WM may play an essential role in mediating music training effects (Moreno and Bidelman, 2014). Unlike short-term memory, WM requires not only temporary storage, but also the processing and manipulation of information (Baddeley, 1992). According to Baddeley and Hitch’s (1974) model, WM consists of two slave systems and a central executive system. The two slave systems—visual-spatial sketchpad and phonological loop—provide the fundamental basis for storing and maintaining visual-spatial and verbal-linguistic information, respectively. The central executive system reflects on domain-general processing and provides a certain workspace for ongoing information manipulation and other cognitive activities. WM refers to a wide range of information processing, including visual-spatial, verbal, and auditory WM. Consequently, many types of WM tests were developed based on the different types of WM and the three processing systems in Baddeley’s (1992) model, which include, for example, the DS forward task, the DS backward task, the matrix span test, the Corsi block span test, the complex span test, and so on (Talamini et al., 2017).

The DS task (Wechsler, 2003) is a valid and commonly used test for measuring verbal WM from both storage and executive perspectives. The test consists of two parts: DS forward and DS backward. The former requires accurate repetition of a presented number sequence, which may represent the component phonological loop in the model. In contrast, the latter requires participants to repeat the numbers in reverse order and, therefore, requires further manipulation of the numbers and executive processing while storing them. Previous research has found evidence of the enhancement of both aspects in adult musicians and musically trained children when compared to untrained individuals. However, the literature is inconsistent regarding which component of the WM is enhanced in musically active individuals. For example, in Suárez et al. (2016), enhanced memory performance in musicians was found in the DS back task, reflecting central executive functions, but not in the DS forward task, reflecting the phonological loop. Similarly, Guo et al.’s (2018) training study of 6–8-year-old children found greater improvement in DS backward scores in the music group than in the control group. In contrast, Saarikivi et al. (2019) investigated the development of WM in children and adolescents aged nine through twenty and reported that musically trained participants outperformed their non-trained peers only on the DS forward test. Similar evidence has been found in other studies (Lee et al., 2007; Hansen et al., 2013). Schellenberg (2011) found that 9–12-year-old children who had music training obtained significantly higher DS total scores than children in the control group, while Virtala et al. (2014) reported that there were only marginally significant differences in DS total scores between adult musicians and non-musicians.

However, the limitations and inconsistencies of implementation were unavoidable in the reported studies. Some studies implemented interventions that may have been too short and, therefore, unable to observe the enhancement. For example, in Guo et al.’s (2018) study, the training sessions lasted for only 6 weeks; in Shen et al.’s (2019) study, the training program duration was 12 weeks. Some studies (Lee et al., 2007; Schellenberg, 2011; Suárez et al., 2016) were cross-sectional and directly compared musically trained and untrained children or adults, which may make it difficult to draw conclusions regarding causation. Furthermore, in some studies, the sample size was relatively small, so the results may not be generalized to a larger population (Fujioka et al., 2006; Virtala et al., 2014; Kumar and Krishna, 2019) or may lead to false-positive results (Button et al., 2013).

Language and music share similar cognitive demands, including auditory, somatosensory, visual, and cross-modal processing. Previous research has suggested that bilingualism also benefits one’s executive functions, especially inhibitory control (Bialystok et al., 2004; Carlson and Meltzoff, 2008; Bialystok and DePape, 2009), as well as WM (Grundy and Timmer, 2017; Antón et al., 2019), although Alain et al. (2018) found that the effect of bilingualism on WM may be supported by different neural activities from those of music expertise. Antón et al. (2019) found that bilingual children outperformed monolinguals on the DS backward but not on the DS forward. The effects of bilingualism reflect a possible role of language processing on more general cognitive functions.

Addressing the limitations of the previous studies above, in our study, we used an randomized controlled trial (RCT) design and investigated the effects of music training on WM performance during a 1-year longitudinal training program in Beijing, China. Over 100 elementary school children were recruited and randomly allocated to music and second-language training groups. Language training was chosen as an active control to investigate the possible unique effect of music training on WM, apart from language learning. A passive control group was also included. To further balance the possible bias between the training groups that may result from dropouts, a propensity score method that is commonly used in medical experiments was applied for the data analysis. We aimed to investigate if and how 1 year of extracurricular group-based music training can benefit school-aged children’s WM compared with language training and no training. In addition, we aimed to determine whether there were other more general effects of music training on children’s cognitive development in terms of verbal and spatial skills.

## Materials and Methods

### Participants

One hundred and nineteen children from 6 to 10 years of age were recruited at the first stage of the study and randomly assigned to the language (*n* = 60) or music (*n* = 59) groups. Nineteen children (fourteen boys) in the music group and seven (four boys) in the language group were unable to attend the courses due to scheduling conflicts. These twenty-six children, along with eleven newly recruited children from the same school, formed the passive control group (*n* = 37). Three children in the music group, three in the language group, and one in the passive control group voluntarily withdrew from the study. This resulted in 123 participants at the baseline stage: fifty in the language group, 37 in the music group, and 36 in the control group. All participants were native Chinese speakers. Twelve children (three in the music group, three in the language group, and six in the control group) failed to attend the training classes and take the post-training tests. Thus, there were 111 participants in the post-test: language group (*n* = 47), music group (*n* = 34), and control group (*n* = 30). In the analysis, outliers were defined as those with baseline scores for forward or backward DS tasks that were more than three standard deviations from the mean. Thus, three participants were identified: two had exceptional scores on the DS forward pre-test, and one had an exceptional score on the DS backward pre-test. Since only one had attended the post-test, 110 participants were included in the analysis: 46 in the language group (23 boys), 44 in the music group (eight boys), and thirty in the control group (twenty-two boys). Table 1 shows additional descriptive statistics.

**TABLE 1:** Descriptive statistics of the background variables in three groups at baseline.

	Music group	Language group	Control group	*df*	*F* or *t*	*p*
	(*n* = 34)	(*n* = 46)*	(*n* = 30)			
Age	8.75 ± 0.78	8.48 ± 0.82	8.57 ± 0.80	107	1.102	0.336
Attendance rate (%)	84.00 ± 20.45	86.65 ± 19.25	–	78	0.593	0.555
WISC-block design	10.26 ± 4.60	9.26 ± 4.31	9.97 ± 4.58	107	0.534	0.588
WISC-vocabulary	12.35 ± 3.32	11.61 ± 3.04	11.83 ± 2.94	107	0.571	0.566
				***N***	**X^2^**	***p***
**Father’s education**						
Higher level (*n*)	17 (50%)	19 (42.2%)	17 (56.7%)	109	1.541	0.463
Lower level (*n*)	17 (50%)	26 (56.5%)	13 (43.3%)			
**Mother’s education**						
Higher level (*n*)	15 (44.1%)	23 (50%)	13 (43.3%)	109	0.579	0.749
Lower level (*n*)	19 (55.9%)	22 (47.8%)	17 (56.7%)			
**Family income**						
Higher income (*n*)	18 (52.9%)	19 (41.3%)	16 (53.3%)	109	1.258	0.533
Lower income (*n*)	16 (47.1%)	26 (57.8%)	14 (46.7%)			
**Gender**						
Boys (*n*)	8 (23.5%)	23 (50%)	22 (73.3%)	110	15.938	<0.0001
Girls (*n*)	26 (76.5%)	23 (50%)	8 (26.7%)			

*The SES scores of one participant in English group was missing, n = 45 when the variable is “family income”, “father education” and “mother education”.

Parents provided written informed consent and were compensated for local transportation and time. - The study was approved by the Institutional Review Board at the State Key Laboratory of Cognitive Neuroscience and Learning, Beijing Normal University, and conducted in accordance with the norms of the Declaration of Helsinki.

### Training Procedure

The training program was based on a large longitudinal study conducted in Beijing (Tervaniemi et al., 2021). The training sessions lasted for two semesters, during which the children received 50 1-h sessions of music/language training after their normal school curriculum. The curriculum of music training combined the Kodaly method with a well-established curriculum for basic knowledge of music, music theory, and solfeggio (Zhao, 2008), which includes fundamental rhythm and pitch skills, sight reading, and singing. Language training taught English as a second language, focusing on English word decoding, phonics, and vocabulary. Teaching materials included relevant textbooks: *Letter Land* (Wendon, 2009; Holt, 2011), *Root Phonics English* (Sun and Lytton, 2010), and *Pandeng English* (Pandeng English Project Team of State Key Laboratory of Cognitive Neuroscience and Learning at Beijing Normal University, 2012). This training protocol has been used in previous studies (Li et al., 2018; Xu et al., 2021). Teachers who were professionally trained in music and English language instruction at the master’s level were hired for this project. A lead teacher always conducted the lesson in each class with an assistant teacher to help children with difficulties and assist with classroom management. During the last session of each semester, a Harvest Festival was held in each class to motivate children’s learning in the classes; those who had studied diligently or performed well received a prize at the festival. Supplementary Table 1 presents information about the fidelity check. The results of the fidelity check, in both the English and music training programs, showed good adherence to the teaching curriculum and plans (ratings above 4.8 on a 1–5 Likert scale). No group differences were observed in the most (first three) categories of fidelity ratings. However, the students’ involvement in music classes was significantly better than in English classes.

Students’ attendance was recorded; the average attendance was higher than 80% (Table 1). During the programs, the children were asked: “Did you generally like the sessions?” They answered using a 5-point Likert scale (1 = *I hate it*; 2 = *I don’t like it*; 3 = *I don’t know*; 4 = *I like it a bit*; 5 = *I like it very much*). In the music group, the mean score for this question was 4.3, and in the language group, it was 4.7, without significant group differences [*t*(56.24) = 1.83; *p* = 0.072; Cohen’s *d* = 0.488].

### Behavioral Measurements

#### Background Questionnaire

Demographic questions included the children’s gender, age, parents’ ages, and the family’s socioeconomic status (SES). Family SES was based on the educational level of both parents, from none to doctoral level. The family’s annual income was also reported by the parents. The data were further divided into two categories according to the respective median: higher family income (above CNY 100,000 annually; approximately USD 15,469) vs. lower family income (less than CNY 100,000) and a higher level of education (above high school) vs. lower education (up to high school). Table 1 shows additional descriptive statistics.

#### Wechsler Intelligence Scale for Child IV

Three subtests were chosen from the Chinese version of the WISC-IV test (Zhang, 2009): DS, block design, and vocabulary. They were conducted before and after the training programs.

***Digit span*** measures short-term auditory memory and WM. The test consisted of the forward DS and backward DS subtests. In the forward span task, the children were presented with a series of numbers and asked to repeat all the numbers in the same order. In the backward span task, the children also heard a series of numbers, but were asked to recall them in reverse order. The number of correctly remembered trials was recorded as the original score for each forward span task and backward span task. The standardized total scores were calculated using Chinese norms (Zhang, 2009).

***Block design*** measures children’s spatial ability. Within a limited time, the participants were asked to assemble blocks to reproduce the given designs, matching the white-and-red design pictured. Each block has two red sides, two white sides, and two sides that are half white and half red. The original scores were the total number of trails in which the children successfully placed all the blocks within the limited time. The standardized total scores were calculated using Chinese norms (Zhang, 2009). The designs were arranged in order of increasing difficulty.

***Vocabulary*** is an untimed verbal core subtest. The test measures verbal fluency, concept formation, and word knowledge and is comprised of twenty-five vocabulary words presented in order of increasing difficulty. The children were asked to explain the meaning of each word. The tasks stopped when the children failed to correctly explain the words. The original scores were obtained from the total number of correctly explained words. The standardized total scores were calculated using Chinese norms (Zhang, 2009).

#### Data Analysis Procedure

In a longitudinal research project with an intervention design, ideally, the groups of participants have balanced background variables to achieve the validity of the between-group comparison. This balance is usually a spontaneous subsequence when randomization is carried out on a sufficiently large sample. However, the small sample of 110 participants in this study (divided into two intervention groups and one control group), as well as the possibility of dropouts, may have caused the risk of imbalanced covariates. Thus, we adopted the propensity score (PS) method (Ho et al., 2007; Hansen and Bowers, 2008) to control for the participants’ baseline characteristics. By using PS, this study was able to create balanced groups in which pairs of participants were similar, except for their experimental statuses, so that the main effect of the intervention could be unbiasedly estimated.

To calculate PS, logistic regression is usually used in this kind of analysis to predict the probability of being in the case group. Then, participants from one experimental group were matched with participants from the other groups on the magnitude of their scores to create groups with balanced covariates. However, research has shown that this procedure can be problematic in studies with small sample sizes. Holmes and Olsen (2010) examined three strategies for smaller sizes (*n* = 112) and recommended that using the PS score as a covariate be the optimal method for analyzing small sample data. Thus, we followed the recommended steps (Leite, 2016) as follows: (1) Estimate the scores—a multinomial logistic regression method was used for estimating the propensity scores. Age, gender as a dummy variable, WISC-block score, and WISC-vocabulary score were included as covariates in the model. The baseline measures were also entered in the model, including either the DS total score, DS forward score, or DS backward score. (2) Calculate the propensity weights—following Leite’s (2016) approach, a propensity weight (PW) for each participant was further obtained by calculating the inverse of each PS. PWs were then assigned to the entire dataset. (3) Evaluation of covariate balance—pre-and post-experiment balancing of confounders between treatment groups, namely, the music group, language group, and passive control group, needs to be checked and reported in PS studies. We made a between-group pairwise comparison for each covariate and calculated the absolute standardized effect size and *p*-value. Effect sizes above 0.25 (Hansen, 2004) or *p*-values below 0.05 (Rubin, 2001) are considered a large imbalance of the covariate.

Finally, an analysis of covariate (ANCOVA) was conducted to estimate the group differences on post-test DS measures, with PW as the covariate (Holmes and Olsen, 2010). Based on previous research, we hypothesized that the effects of music training may differ on forward and backward tests (Saarikivi et al., 2019), so we analyzed the DS data separately for the forward and backward scores. Further multiple comparisons between groups were performed using the Bonferroni adjustment.

The data analysis was conducted in R (R Core Team, 2020). The *vglm* function in the “VGAM” package (Yee, 2020) and the *bal.stat* function in the “twang” package (Cefalu et al., 2021) were used to conduct multinomial logistic regressions and to assess the imbalance of the confounding variables, respectively. The function *emmeans* in the package “emmeans” (Lenth, 2020) was used for the *post hoc* test in the ANCOVA.

## Results

Table 1 shows the demographic variables for the three groups in terms of age at baseline, SES, baseline IQ, and attendance rate. No other significant differences were found in these variables among the three groups except for gender distribution—there were more boys in the control group than in the music group. This imbalance was caused by the selective participation of boys in the activities: despite random allocation, of the twenty-six participants who did not want to join the experimental (music, language) groups but who went to the passive control group, nineteen were boys.

We applied PS analysis to balance the bias from gender and other baseline measures. Table 2 summarizes the pairwise covariate balance before and after adjusting with the PW from multinomial logistic regression, as well as the unadjusted balance in the baseline for DS total, DS forward, and DS backward, respectively. An effect size of 0.25 or greater is considered large (Hansen, 2004). As demonstrated in the table, the weights obtained with the multinomial logistic regression models provided a good covariate balance and were used in the final analysis.

**TABLE 2:** Summary of covariate balance.

	Group 1	Group 2	Method	Maximum standardized effect size	*P*-value
DS total	Music	English	Unadjusted	**0.58**	0.12
	Music	Control	Unadjusted	**1.36**	**0.01**
	English	Control	Unadjusted	**0.98**	0.18
	Music	English	MLR	0.12	0.63
	Music	Control	MLR	0.24	0.33
	English	Control	MLR	0.12	0.33
DS forward	Music	English	Unadjusted	**0.58**	**0.01**
	Music	Control	Unadjusted	**1.45**	0.25
	English	Control	Unadjusted	**1.3**	0.63
	Music	English	MLR	**0.26**	0.33
	Music	Control	MLR	**0.41**	0.15
	English	Control	MLR	0.16	0.15
DS backward	Music	English	Unadjusted	**0.7**	**0.02**
	Music	Control	Unadjusted	**1.23**	** < 0.001**
	English	Control	Unadjusted	**0.67**	0.46
	Music	English	MLR	0.21	0.46
	Music	Control	MLR	0.18	0.45
	English	Control	MLR	0.1	0.45

*1. GBM, general boosted model; MLR, multinomial logistic regression; 2. Numbers in **bold** indicates the standardized effect size over 0.25 or p-value below 0.05 indicating imbalance on those covariates. Background variables include age, attendance rate, SES, gender, and general baseline cognitive abilities. For SES, “higher level” is defined as the reported education level or family income was higher than the median; “lower level” is defined as the reported education level or family income was lower than the median.*

**FIGURE 1:**
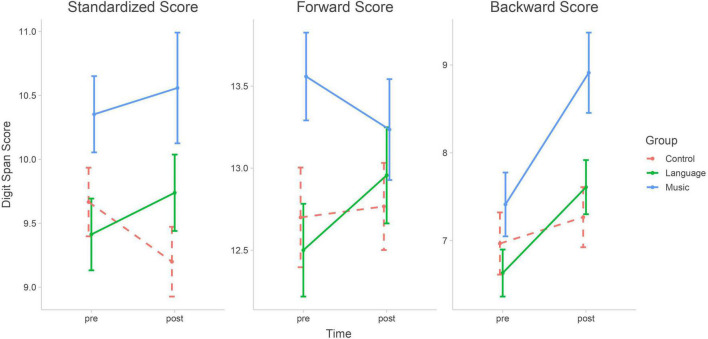
Comparisons of digit span scores between groups (Mean and SE). Music group gained significant improvement compared with Language and Control group in digit span backward scores. However, no significant interaction between Group and Time was found in the digit span forward scores or digit span standardized score.

Figure 1 shows a comparison of the DS scores among the three groups. The one-way ANCOVA was conducted to determine the group differences in the DS score in the post-test, after controlling for the propensity weights in the pre-test. Thus, the effects of the intervention can be estimated after controlling for prior differences. For the standardized DS total score, the results showed there was a significant group effect after controlling for the propensity weights in the pre-test [*F*(2, 106) = 3.598, *p* = 0.031]. However, the *post hoc* test showed that there was a significant difference between the music and passive control groups (*p* = 0.029) but no significant differences between the music and language groups or between the language and passive control groups (*p* > 0.05).

Next, we analyzed the outcome of the forward and backward subtests separately to reveal whether music training affected the phonological loop reflected by the forward subtest or the central executive system reflected by the backward subtest. As our main finding, we identified a discrepancy between the DS forward and backward raw scores as follows. For DS forward raw scores, the one-way ANCOVA showed no difference between the groups after controlling for the propensity weights in the pre-test [*F*(2, 106) = 0.583, *p* = 0.560]. In contrast, for DS backward raw scores, the one-way ANCOVA showed a significant group effect after controlling for propensity weights in the pre-test [*F*(2, 106) = 5.038, *p* = 0.008]. The music group outperformed the passive control group (*p* = 0.013) and the language group (*p* = 0.039); there were no differences between the language group and the passive control group (*p* = 0.14).

The other two measures—block subtest scores and vocabulary subtest scores—were analyzed with the same procedure as PS analysis. The one-way ANCOVA with the propensity weights as covariates showed that there were no significant group differences on either the block subtest score [*F*(2, 106) = 0.464, *p* = 0.630], or the vocabulary subtest score [*F*(2, 106) = 0.593, *p* = 0.554].

## Discussion

The aim of our study was to investigate the effects of music training on auditory WM in school-aged children. The results revealed different effects of interventions, namely music training, language training, and no training, on the performance of DS tasks. On the general performance of the DS task, the musically trained group showed significant superiority compared to the control group after controlling for prior bias before the training and the baseline level of the DS performance. However, this superiority was observed only in the DS backward performance. Regarding the DS forward performance, no such difference was found between the groups.

This result is in line with previous research indicating that DS forward and DS backward reflect different cognitive functions. Reynolds (1997), using factor analysis, found that forward and backward tasks indicate two distinct memory processes. Furthermore, in a study investigating attention deficits and DS performance, only DS backward scores predicted children with attention deficit hyperactivity disorder, while the DS forward task did not (Rosenthal et al., 2006). Our results support the view that DS forward and DS backward are distinct, measuring different cognitive processes—DS forward involves short-term auditory memory processes, whereas DS backward involves additional components of attention and executive functions.

Our results show that music training may be more beneficial for attention and executive memory processes, which is indicated by enhanced DS backward scores. This supports previous findings of positive associations of music expertise with the DS backward task (Guo et al., 2018) and higher cognitive functions, such as WM (Roden et al., 2014; D’Souza et al., 2018) and other executive functions (Degé et al., 2011; Saarikivi et al., 2016; Jaschke et al., 2018; Shen et al., 2019).

Notably, the negative results of the DS forward test were discrepant with previous findings. George and Coch (2011) found that DS forward scores were positively correlated with years of music training. Accordingly, Saarikivi et al. (2019) found that musically trained children and adolescents outperformed their untrained peers in DS forward but not DS backward tasks. They argued that music training may benefit WM, specifically in retaining and reproducing auditory sequences rather than in updating information in the mind. However, in the current study, music training did not produce a significant improvement in maintaining information indexed by the DS forward tasks.

One possible reason may be that this results from having a language background than in the majority of the literature---the participants in previous studies were speakers of non-tonal languages, whereas in the present study the spoken language is Chinese mandarin regarded as tonal language.^1^ Bidelman et al. (2013) found that speakers of Cantonese, a tonal language, outperformed speakers of non-tonal languages on the tonal memory task, in which participants were asked to judge whether the probe tone was present in a four-tone sequence they had heard before.

In this study, the digit sequences in Mandarin, which is a tonal language, always have the same tones, and these tones may sound like melodies to children. The daily experience of listening and speaking melodic sentences may equip children with better auditory memory than the non-tonal language speakers, even without music training. While the performance of the DS forward task consequently benefited from the tonal melodies created by the digit sequences, the children might have already possessed a good level of memory for the DS forward, and music training may not be beneficial comparatively. The DS forward score in the music group might have dropped slightly because of the random fluctuation in the children’s performances. However, when the task was to list numbers in reverse order for the DS backward task, this melodic cue of the digit sequences was no longer helpful.

Another difference between our earlier findings and the literature can be found in the type of music training. While the training in this study was group-based and given as extracurricular lessons to schoolchildren, in previous studies, the musically trained participants were involved in instrumental training programs. Consequently, the discrepancy in the results may be explained by different demands of the given training; individual lessons emphasized fine-grained auditory functions, while group-based lessons in our study focused on acquiring music knowledge—for example, the recognition and classification of rhythm patterns and melodies, as well as interactions with teachers and peers. Thus, attention and executive functions might be practiced more than in other programs.

Next, we discuss the limitations of our study. Our initial purpose was to randomly assign the children to groups. However, there was a high dropout rate before the onset of the training program—several children dropped out of classes because of “scheduling conflicts.” This might have led to an initial group difference before the training in the DS task but interestingly not in the block design and vocabulary task. It turns out that motivation and other environmental “hidden factors,” such as school achievements and parents’ personalities and parenting styles, may become critical barriers during random assignments (Schellenberg, 2020). When there was a weak commitment from the participants, those less motivated tended to choose other activities instead of staying in the classes.

However, if this issue were considered, what would happen if the less-motivated children were forced to stay and participate in the music group lessons? In addition to being unethical, it might still lead to an imbalance in motivation across groups, which could also impact the training effect. Moreover, some researchers have argued that randomization and the inference of causality are complicated. The group difference might still be present because of either gene-influenced individual differences or environmental factors, even if they were absent before the training (Schellenberg, 2020). Therefore, while solving the practical challenges of random assignment in a study, more factors, such as individual and familial background, should also be considered during the design, observation, and analysis processes of a training study in children.

In sum, we found that group-based music training enhanced children’s auditory WM in terms of the executive system, as indexed by the DS backward test. In contrast, there was no evidence of the enhancement of simple storage of the digit WM, as indexed by the DS forward, resulting from music training. This could be due to the native tonal language background of the children, which may help their phonological storage with or without music training. To conclude, our results indicate that music training may enhance children’s ability to manipulate information as a higher-order cognitive process, but not their simple storage capacity of auditory information.

## Data Availability Statement

The statistical data supporting the conclusions of this article will be made available by the authors, without undue reservation.

## Ethics Statement

The studies involving human participants were reviewed and approved by Institutional Review Board at the State Key Laboratory of Cognitive Neuroscience and Learning, Beijing Normal University. Written informed consent to participate in this study was provided by the participants’ legal guardian/next of kin.

## Author Contributions

MT and ST designed the research plan. PN, CW, BD, SL, and JL monitored the training programs and conducted the research under the mentorship of ST. PN analyzed the data, wrote the initial draft of the manuscript, and prepared the data figures. GR helped with the data analysis and results reporting. All authors contributed to the revision of the manuscript and accepted the final version of it.

## Conflict of Interest

The authors declare that the research was conducted in the absence of any commercial or financial relationships that could be construed as a potential conflict of interest.

## Publisher’s Note

All claims expressed in this article are solely those of the authors and do not necessarily represent those of their affiliated organizations, or those of the publisher, the editors and the reviewers. Any product that may be evaluated in this article, or claim that may be made by its manufacturer, is not guaranteed or endorsed by the publisher.

## Footnotes

1 Here, tonal language (Chinese, Vietnamese, Thai, etc.) is one in which the same series of sounds can have different semantic meanings depending on the tones (pitch) of the word. In contrast, in non-tonal languages (English, Spanish, etc.), the word’s meaning is not influenced by pitch.
