# Supplementary material for: Effects of Music Training on the Auditory Working Memory of Chinese-Speaking School-Aged Children: A Longitudinal Intervention Study
Source: Front Psychol. 2022 Jan 26;12:770425. doi: 10.3389/fpsyg.2021.770425 (PMC8825862; doi:10.3389/fpsyg.2021.770425)
Supplement: Supplementary file 1 [file Table_1.docx]

Supplementary material:

| Table I group differences on fidelity check | | | | | | |
| --- | --- | --- | --- | --- | --- | --- |
|  | English class | Music class | T | df | p | d |
| teaching curriculum matched | 4.9900 | 5.0000 | -1.000 | 99.000 | .320 | -0.201 |
| teaching content completed | 4.9700 | 5.0000 | -1.347 | 99.000 | .181 | -0.271 |
| implementation matched | 4.8400 | 4.9100 | -1.167 | 190.128 | .245 | -0.169 |
| students' classroom involvement (how many times on average each child interacts with teachers in one session) | 1.8050 | 2.0950 | -2.047 | 177.857 | .042 | -0.307 |
